# Mini-hearts for disease modeling and drug testing – process optimization versus biological functionality

**DOI:** 10.3389/fbioe.2025.1719533

**Published:** 2026-01-05

**Authors:** Jana Hecking, Mariel Cano-Jorge, Robert Passier, José Manuel Rivera-Arbeláez

**Affiliations:** 1 Applied Stem Cell Technologies, Department of Bio Engineering Technologies, TechMed Centre, University of Twente, Enschede, Netherlands; 2 Department of Anatomy and Embryology, Leiden University Medical Centre, Leiden, Netherlands; 3 BIOS Lab-on-a-Chip Group, MESA+ Institute for Nanotechnology, Max Planck Institute for Complex Fluid Dynamics, University of Twente, Enschede, Netherlands

**Keywords:** tissue engineering, 3D cardiac models, engineered cardiac chambers, drug testing, disease modeling

## Abstract

Traditional two-dimensional cell cultures and *in vivo* animal studies fail to fully recapitulate human cardiac physiology, highlighting the urgent need for more relevant human-based models. Engineered three-dimensional cardiac systems - including organoids, engineered heart tissues, and heart-on-chip platforms offer promising alternatives, providing structural and functional insights into cardiac biology. However, a critical limitation of these models is their inability to perform fluid pumping and relaxation, which together define fundamental heart function. Engineered cardiac chambers have emerged to address this gap, enabling physiologically relevant pressure-volume measurements and capturing both contractile and diastolic dynamics that mimic aspects of native cardiac hemodynamics. This mini-review examines the current state of engineered cardiac chambers and highlights their main design features. We discuss their applications in disease modeling and drug testing, and outline key factors influencing the optimization of these models, including balancing biological fidelity with process efficiency through modular design principles. Overall, engineered cardiac chambers represent a unique, powerful platform to improve mechanistic understanding of cardiac disease, offering significant potential to advance cardiovascular research and therapeutic development.

## 1 Introduction

The human heart is a vital organ responsible for pumping blood throughout the body, delivering oxygen and nutrients to tissues and removing metabolic waste products. Cardiovascular Diseases (CVDs) encompass a spectrum of medical conditions related to the heart and the vascular system. These diseases represent a significant health challenge, being the leading cause of mortality and disability worldwide. In 2017 alone, CVDs accounted for 17.8 million deaths globally, a trend that continues to rise (Mensah et al., 2019).

This alarming mortality rate can be partly attributed to substantial hurdles within the drug development pipeline, where approximately 90% of drug programs fail, primarily in late stage clinical trials (Marshall et al., 2018). A major contributor is the limited predictability of preclinical studies, often reliant on Two-dimensional (2D) *in vitro* platforms and *in vivo* animal models. While 2D models offer high throughput and simplicity, they fail to replicate physiological complexity (Ayuso et al., 2021). *In vivo* testing, despite providing a comprehensive organism-level response, is hindered by physiological discrepancies between species, ethical concerns, and high costs (Ayuso et al., 2021; Shanks et al., 2009). Thus, there is a great need for more predictive human-based cardiac *in vitro* models to enhance CVD therapeutic strategies and avoid drug-induced cardiotoxicity (Marshall et al., 2018; Muniyandi et al., 2023).

Various types of engineered Three-Dimensional (3D) cardiac models, often derived from human Pluripotent Stem Cells (hPSCs) are currently under development: Organoids, Engineered Heart Tissues (EHTs), and Heart-On-Chip (HOC) models each offer distinct advantages (Ronan et al., 2023; Liu et al., 2023). Cardiac organoids represent self-organizing 3D structures used to study fundamental aspects of embryonic development and congenital heart diseases (Hofbauer et al., 2021; Schmidt et al., 2023; Lewis-Israeli et al., 2021). EHTs, tissue strips typically positioned between two flexible micropillars, are ideal for examining cardiac contractile performance based on pillar deflection upon beating (Ronan et al., 2023; Liu et al., 2023; Windt et al., 2023; Ribeiro et al., 2022; Mannhardt et al., 2016; Hansen et al., 2010; Turnbull et al., 2014). HOC models are positioned at the intersection of microfluidics and tissue engineering. These intricate systems are known for their ability to modulate and precisely control both biochemical cues and physiological or pathophysiological parameters (Liu et al., 2023; Li et al., 2022). However, a critical limitation of the described cardiac models is their inability to pump fluid (the heart’s main function). To address this, 3D Engineered Cardiac Chambers (ECCs) or also called engineered ventricles have emerged. These models more closely resemble the structural architecture of the heart, pump fluid, allow for analysis of Pressure-Volume (PV) readouts, facilitate control of preload and afterload, and are exposed to multiaxial stress (Li et al., 2018; MacQueen et al., 2018). Thus, the unique features of ECCs provide a novel opportunity for drug testing and disease modeling, especially in the context of complex cardiac diseases.

This mini-review provides an overview of existing ECCs, with a focus on key features such as fabrication process, cell types and density, throughput capabilities, PV readouts, and their current applications in disease modeling and drug testing. After describing the essential considerations for utilizing ECCs in this context, we discuss the application potential of existing platforms and identify opportunities for future models, highlighting how they may effectively contribute to advancements in both disease modeling and drug testing.

## 2 Existing engineered cardiac chambers

Early advancements in the development of ECCs primarily aimed at creating cardiac pouches for transplantation purposes. For example, Yildirim et al. developed a biological ventricular assist device using Neonatal Rat Ventricular Myocytes (NRVMs), which exhibited spontaneous beating (Yildirim et al., 2007). Similarly, Gonen-Wadmany et al. reported a chamber-like bioartificial cardiac muscle characterized by mechanical strain-mediated cell organization (Gonen-Wadmany et al., 2004). Although these early models lack hemodynamic metric measurements such as PV readouts, they paved the way for further innovations in the field. Moreover, efforts in heart decellularization and recellularization, leverage natural extracellular matrix to recreate functional hearts (Hochman-Mendez et al., 2022; Yasui et al., 2014; Garreta et al., 2016). However, this method is unsuitable for drug testing and disease modeling due to the extremely high cellular demands. Accordingly, this review will focus on ECC models with potential for drug testing and disease modeling.

### 2.1 Focus features in existing cardiac chambers

The first ECC model designed for this application and capable of *in vitro* hemodynamic measurements was introduced by Lee et al. (2008). More ECCs were developed across various laboratories with different fabrication strategies, each offering unique advantages (focus features). Fabrication strategies include molding, scaffold seeding, 3D bioprinting, and cell sheet wrapping (Table 1). Focus features are often tailored toward enhancing physiological relevance and complexity to more closely emulate the native heart. Key focus areas include perfusion, valve integration, Cardiomyocyte (CM) alignment, complex geometries, functional readouts, and specific applications (Table 1).

**TABLE 1:** Overview of existing cardiac chambers with focus feature, fabrication method, cell type and number, throughput, pressure-volume readouts, and drug testing and disease modeling applications divided by research lab.

Lab	Focus feature	Fabrication method	Cell type and number	Through-put	PV readouts		Drug test. and disease mod.	References
Costa	Drug testing and disease model applications	Molding with outer agarose mold and inner catheter balloon (changed to permanent balloon)	15M NRVMs and 1M NR cardiac FBs per mL	1	Via catheteri-zation	ΔP = 1–2 mmH_2_O; EF = 3–5%	Cryoinjury model, positive inotropic response	Lee et al. (2008)
			10M hESC-CM and 1M human (dermal/foreskin) FBs per tissue			ΔP = 1.26 ± 0.12 mmH_2_O; EF = 2.44 ± 0.27%	Positive and negative inotropic and chronotropic response, FRDA model, HFpEF model	Li et al. (2018), Roberts et al. (2022), Keung et al. (2019), Wong et al. (2019), Costa et al. (2024)
Birla	Perfusion and valve	Cell injection into molded chitosan scaffold and cell patch	10M NRVMs per scaffold and 2M primary cardiac cells per patch	3 (individual seeding)	Via catheterization; ΔP = ∼3 mmHg; ΔP with valve = 0.05–0.12 mmHg		-	Patel and Birla (2017), Patel and Birla (2018)
Parker	Valves	Nanofiber pull spun scaffolds and cell seeding	12M NRVMs or hiPSC-CMs per ventricle scaffold	1	Via catheterization without valves with hiPSCs; ΔP = ∼50 μmHg; EF = ∼0.2%		positive chronotropic response, structural arrhythmia model	MacQueen et al. (2018)
	Cell alignment and complex geometries	rotary yet spinning of scaffold with cell seeding	5–7M NRVMs and 10–14M hiPSC-CMs per ventricle scaffold		Via PIV in open single chambers	EF = 3.3 ± 1.7%	-	Chang et al. (2022)
		3D printing of scaffold with fiber infused ink and cell seeding	5M NRVMs and 8M hiPSC-CMs per ventricle scaffold			EF = 5.94 ± 1.66% (with hiPSCs)	-	Choi et al. (2023)
Fein-berg	Cell alignment and complex geometries	Dual-material 3D-bioprinting with collagen ink and cell ink using FRESH method	hPSC-CMs and 2% cardiac FBs (numbers not known)	1	-		-	Lee et al. (2019)
Ogle	Perfusion and complex geometries	3D bioprinting with *in-situ* hiPSC-CM differentiation	15M hiPSCs per mL	1	Via catheterization; ΔP = ∼0.2 mmHg; EF = ∼0.7%		positive and negative inotropic and chronotropic response	Kupfer et al. (2020)
Radisic	Cell alignment and perfusion and conical shape	Wrapping 2D microfabricated cell sheets	6.9M NRVMs per cell sheet	1	Via catheterization at 1 Hz stimulation; ΔP = ∼2.8 mmHg; EF = 2%		-	Mohammadi et al. (2022)
Chen	Miniaturization, valves and control of afterload	Nanoscale-resolution scaffold by two-photon direct laser writing and cell seeding	170 k hiPSC-CMs and 4 k primary human bone marrow stromal cells per ventricle scaffold	1	Via PIV and FEM simulations; ΔP = 0.2145 mmHg; EF = ∼4.06%		-	Michas et al. (2022)
Matsu-saki	Complex geometries	3D printing of collagen scaffold in microgel support bath and cell seeding	10M hiPSC-CMs per construct	1	-		-	Xie et al. (2023)
Engel	Complex geometries	3D bioprinting in microparticle support bath with hiPSC-CMs	25M hiPSC-CMs per mL	1	-		positive chronotropic response	Esser et al. (2023)
Fischer	Measure pulsatile flow via sensor	Cell seeding on biofunctional PDMS-membrane in seeding reactor and culture in bioreactor	∼750 k hiPSC-CMs (and 50 k hCFs or 10 k hCMEC) per cm^2^	1	Via flow sensor; CO = 1.2 μL/min; SV = 13.8 nL		positive and negative inotropic and chronotropic response	Kuckelkorn et al. (2025)
Kim	Cell alignment	Cell seeding on flexible nanofabricated sheets, wrapping, molding and casting	∼180 k hiPSC-CMs and hiPSC-ECs (7:1) per cm^2^ (double-seeded)	1	Via catheterization; ΔP = ∼0.5 mmHg		-	Williams et al. (2025)
Passier	Non-invasive functional readouts	Molding with outer and inner thermo-responsive gelatin molds in custom bioreactor	∼4.5M hiPSC-CMs and ∼400 k hCFs	1	Via imaging of fluid ejection and pressure estimation; ΔP = 0.5 ± 0.1 mmHg; EF = 2.88 ± 0.5%		positive and negative inotropic response	Ribeiro et al. (2025)

Perfusion remains a critical and underrepresented feature, essential when trying to separately control preload and afterload. While Patel and Birla present a single chamber with an inlet and outlet (Patel and Birla, 2017), the Kupfer et al. model stands out by using bioprinting to create a more complex perfusable structure, although it lacks valve integration (Kupfer et al., 2020).

Valve integration has been explored in a few models to replicate unidirectional pumping function. For instance, Patel and Birla initially included a tri-leaflet valve but did not demonstrate unidirectional ECC flow (Patel and Birla, 2017; Patel and Birla, 2018). In contrast, Michas et al. and Macqueen et al. successfully incorporated valves into microfluidic and bioreactor platforms, respectively, with Michas et al. demonstrating unidirectional flow and MacQueen et al. reporting improved PV loop morphology as a result of valve presence (Michas et al., 2022; MacQueen et al., 2018).

CM alignment is crucial to achieve enhanced structural organization and contractile function. Alignment is a central focus in several models, with multiple strategies employed to achieve this. One approach involves the use of nanostructured scaffolds - either rotary jet spun or 3D printed - to guide alignment, with functional outcomes shown to vary based on scaffold architecture (Chang et al., 2022; Choi et al., 2023). For example, Chang et al. demonstrated that differences in fiber angle can significantly influence cardiac function (Chang et al., 2022). Similarly, Lee et al. used 3D printing to control alignment direction and showed wave propagation along the printing axis using calcium imaging (Lee et al., 2019). Another strategy involves patterning of CMs on 2D substrates, which are subsequently wrapped into 3D structures to achieve alignment at defined angles (Williams et al., 2025; Mohammadi et al., 2022).

Complex geometries like two or four-chambered models are often fabricated via 3D bioprinting with cell inks or scaffold printing using materials such as collagen or gelatin-alginate, sometimes embedded in microgel support baths (Chang et al., 2022; Choi et al., 2023; Lee et al., 2019; Kupfer et al., 2020; Esser et al., 2023; Xie et al., 2023). Some of these more complex engineered cardiac chambers lack functional readouts, thus, limiting their use for drug testing and disease modeling (Esser et al., 2023; Xie et al., 2023; Lee et al., 2019). Nonetheless, Kupfer et al., Chang et al. and Choi et al. reported volumetric performance data, with Kupfer et al. additionally demonstrating Pressure Difference (ΔP) measurements (Kupfer et al., 2020; Chang et al., 2022; Choi et al., 2023).

Functional readouts are a priority in several models; notably, Kuckelhorn et al. uniquely integrated a flow sensor, and Ribeiro et al. visualized fluid pumping through a connected glass capillary, thus enabling non-invasive PV analysis (Kuckelkorn et al., 2025; Ribeiro et al., 2025) (Table 1).

## 3 Drug testing and disease modeling in cardiac chambers

### 3.1 Established drug testing and disease modeling in cardiac chambers

Several ECCs have already demonstrated initial applications in drug testing and disease modeling (Table 1). These include chronotropic and/or inotropic responses - both positive and negative - to cardioactive compounds such as isoproterenol, nifedipine, vasopressin, carbachol, and phenylephrine (Lee et al., 2008; Li et al., 2018; Keung et al., 2019; MacQueen et al., 2018; Kupfer et al., 2020; Esser et al., 2023; Kuckelkorn et al., 2025; Ribeiro et al., 2025).

Notably, the work from Costa’s laboratory has been a major driver in advancing ECC disease modeling. In their earliest report, Lee et al. employed cryoinjury to simulate myocardial infarction, demonstrating proof-of-concept evidence of reduced chamber pressure and contraction strength 15 min post-injury (Lee et al., 2008). Subsequent studies expanded these capabilities to model Friedreich’s ataxia, a condition characterized by hypertrophic cardiomyopathy and impaired relaxation (Wong et al., 2019) and, more recently, Heart Failure with preserved Ejection Fraction (HFpEF) (Wong et al., 2019; Costa et al., 2024). The HFpEF ECC model exhibited increased passive chamber stiffness and a leftward-shifted diastolic pressure–area relationship, while maintaining Ejection Fraction (EF), thus recapitulating hallmark features of the disease phenotype. The model was subsequently used to evaluate a novel candidate gene therapy in conjunction with Astra Zeneca (Costa et al., 2024). Interestingly, Costa’s ECC technology, currently at Technology Readiness Level (TRL) level 7, has been transferred to Novoheart for commercial usage.

An additional disease model, a structural arrhythmia model induced by cryoinjury, was described by MacQueen et al. (2018). This approach produced pinned spiral wave patterns that are consistent with clinical arrhythmia phenotypes. Despite these advances, many diseases remain unaddressed and more comprehensive functional and structural characterization of existing disease models is still required. Consequently, the field presents substantial opportunities for developing new ECC-based disease models. The following section will outline key considerations for establishing these models.

### 3.2 Important considerations about drug testing and disease model applications in cardiac chambers

While some ECC models are designed with the ultimate goal of tissue regeneration and eventual implantation, this vision requires incorporating features that more closely replicate the structure and function of the native human heart (Yildirim et al., 2007; Patel et al., 2017; Esser et al., 2023). Although such a long-term objective is scientifically compelling, it remains distant given that major research and economical challenges - such as achieving physiological size, incorporating the full diversity of mature cardiac cell types, and establishing functional vascularization - have not yet been addressed in current systems. In the near term, the most promising and practical applications of these advanced models are in drug testing and disease modeling. This focus aligns with broader regulatory developments, as reflected in the Food and Drug Administration (FDA) Modernization Act 2.0 and 3.0, which promote the adoption of Organ-On-Chip (OOC) platforms and other New Approach Methodologies (NAMs) as alternatives to traditional animal testing (Carratt et al., 2024; Zushin et al., 2023; Ahmed et al., 2023).

**FIGURE 1:**
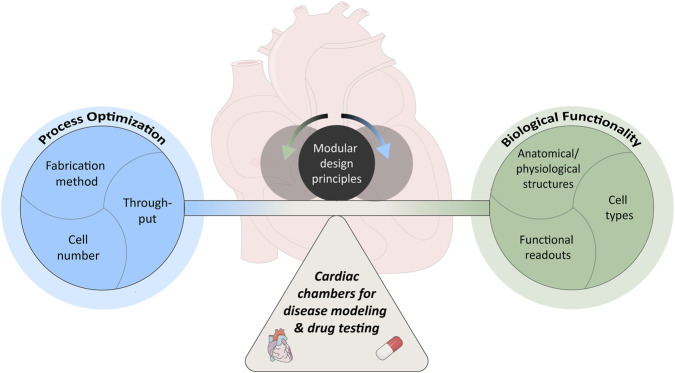
ECC paradigm. Trade-off between focusing on process optimization (blue) and biological functionality (green) when designing ECC models for disease modeling and drug testing applications. Modular design principles (charcoal) can act as regulator to keep the two in balance.

In drug testing and disease modeling applications of ECCs, maximal anatomical fidelity is not the primary requirement. Instead, developers must navigate the trade-off between process optimization (making workflow more cost-effective, reliable and streamlined) and biological functionality (performing intended role in health and disease scenarios) (Figure 1). In this context, process optimization aims to improve the efficiency and scalability of disease modeling and drug testing workflows. While this is a clear priority for commercial applications, it is equally important in academic research to enable meaningful cross-study comparisons, enhance reproducibility, and support cost-saving strategies. A key component of process optimization is the fabrication procedure. Ideally, fabrication should be as simple, rapid, and reproducible as possible, minimizing the number of manual steps to reduce variability and human error (Ma et al., 2021). The process should integrate seamlessly into the broader cell culture pipeline, allowing automated or semi-automated handling (Probst et al., 2018). Standardization of materials, protocols, and device geometries is essential to ensure consistent performance across laboratories and to enable regulatory acceptance (Piergiovanni et al., 2021a; Piergiovanni et al., 2021b). Higher throughput across the entire fabrication, culture and analysis pipeline accelerates screening campaigns and increases statistical power (Probst et al., 2018). The number of cells required per construct directly impacts production cost and scalability. This has driven a trend toward miniaturization in OOC systems, enabling reduced cell input without sacrificing essential functionality (Michas et al., 2022).

Achieving biological functionality and complexity, critical for developing physiologically relevant models, requires deliberate consideration of the cellular composition, anatomical structures, and functional readouts incorporated into the system. One foundational aspect is the inclusion of appropriate mature cell types (Low et al., 2021; Wnorowski et al., 2019). Cardiac pathophysiology often arises from interactions between CMs, fibroblasts (FBs), endothelial cells, smooth muscle cells, and other supporting cell types (Deb, 2014; Tan et al., 2024). In certain disease contexts, reproducing the geometry and architecture of specific components of the heart is essential (i.e., valves in valvular heart disease). Beyond modeling the myocardium, recapitulating the layered architecture of the native heart, including endocardium and epicardium, can be particularly important, as each layer contributes distinct functions (Quijada et al., 2020; Cao and Poss, 2018). Incorporation of valves is critical for reproducing physiologic PV loops and ensuring unidirectional flow (MacQueen et al., 2018; Michas et al., 2022). Blood vessels serve a dual role as they supply oxygen and nutrients to thicker engineered tissues and they participate directly in disease processes such as ischemia (Severino et al., 2020). Importantly, vascularization alone is not sufficient, as active perfusion is required to ensure both the delivery of nutrients and the removal of waste products, adding another layer of complexity. Finally, the inclusion of functional readouts is indispensable. PV-related measures like Cardiac Output (CO) and EF stand out as a unique feature in ECC models over simpler 3D cardiac models. Here, the goal is not necessarily to reproduce physiologic values, but rather to model the desired response in a particular context-of-use. By measuring pressure and volume simultaneously, these models can capture load-dependent and load-independent indices of cardiac performance, making them exclusively powerful (over other *in vitro* 3D models) for investigating systolic dysfunction, diastolic dysfunction as well as overload conditions (Hieda and Goto, 2020; Litwin and Grossman, 1993). Maintaining the balance between biological functionality and process optimization is inherently challenging, as overemphasizing one inevitably compromises the other (Figure 1). A practical strategy to address this is the adoption of modular design principles, enabling the tailoring of model features to the specific context-of-use - such as incorporating only those structural, cellular, and functional elements most critical for faithfully recapitulating the disease or physiological condition under investigation.

## 4 Discussion

Over the past decade, the landscape of *in vitro* cardiac modeling has undergone substantial evolution, moving beyond traditional animal models toward advanced 3D platforms. Among these, ECC models represent the most visually and functionally physiological constructs, capturing hallmark features of the native heart such as coordinated fluid pumping and the generation of clinically relevant hemodynamic metrics. While their design objectives vary, many aim to more closely mimic the structural and functional attributes of the human heart, thus, making functional performance more physiological. Although long-term aspirations include applications in transplantation and tissue regeneration, the most impactful near-term uses are in disease modeling and drug testing, particularly in light of the growing regulatory support for NAMs, such as OOC platforms (Carratt et al., 2024; Zushin et al., 2023; Ahmed et al., 2023).

A central challenge in developing ECC models manifests in balancing process optimization with biological functionality. Process optimization is a critical determinant of whether ECC models can be scaled for meaningful application in both academic and commercial contexts. One key factor is the fabrication method. Simpler approaches, such as molding, allow relatively straightforward and scalable generation of tissues, but rely heavily on self-organization processes, which may introduce variability in final architecture and function (Lee et al., 2008; Li et al., 2018; Ribeiro et al., 2025). More sophisticated fabrication strategies, including nanofabrication and 3D (bio)printing, offer greater control over geometry and microstructure but raise concerns about efficiency, cost, and throughput (Lee et al., 2019; Kupfer et al., 2020; Esser et al., 2023). In addition, these methods oftentimes involve scaffolds which might interfere with tissue properties (Patel and Birla, 2018; Chang et al., 2022; Choi et al., 2023; MacQueen et al., 2018; Michas et al., 2022; Xie et al., 2023; Williams et al., 2025). Therefore, developers need to decide what fabrication choices to prioritize in their context-of-use. Throughput remains an underdeveloped aspect across current models. To date, no published cardiac chamber platform has been designed for parallelized or high-throughput production; most are limited to fabricating a single ECC at a time. While this may be acceptable for early-stage research, it represents a substantial bottleneck for translation into commercial drug screening pipelines. The number of cells required per construct is another important aspect towards scalability. Existing models span a wide range - from approximately 174,000 cells per ECC to about 15 million cells - directly influencing both production cost and feasibility (Lee et al., 2008; Chang et al., 2022; Kupfer et al., 2020). The miniaturized approach described by Michas et al. is currently the most cell-efficient (with 174,000 cells per ECC), representing a cost-saving strategy that aligns well with the broader OOC trend toward minimizing input material while retaining essential functionality (Michas et al., 2022).

Biological functionality, by contrast, prioritizes the physiological fidelity of the model. Several ECCs are derived from human induced Pluripotent Stem Cells (hiPSCs), commonly differentiated into CMs and FBs (Li et al., 2018; Michas et al., 2022; Kuckelkorn et al., 2025; Ribeiro et al., 2025). However, restricting models to only CMs and FBs may overlook crucial cell interactions in diseases like myocardial infarction (Deb, 2014; Tan et al., 2024). Using hiPSCs offers advantages for patient-specific modeling, particularly in the context of genetic disorders, yet still faces the challenge of incomplete cellular maturation and differentiation variability (Wnorowski et al., 2019; Low et al., 2021; Devalla and Passier, 2018). Notably, ECCs have demonstrated a higher degree of maturation than simpler 3D cardiac constructs with increased expression of cardiac proteins and increased sensitivity to positive inotropes, which may improve their ability to recapitulate disease phenotypes (Li et al., 2018; Cheng et al., 2023). Anatomically, select models have incorporated functional valves, producing more physiological PV loops (MacQueen et al., 2018; Michas et al., 2022). However, certain key features remain absent: perfusable blood vessels, although demonstrated in simpler cardiac constructs, have not yet been integrated into chamber systems (Arslan et al., 2023). Likewise, the layered architecture of the native heart (myocardium, endocardium, epicardium) has yet to be reproduced.

Functional readouts remain a defining strength of ECCs. Many platforms employ pressure and/or volume measurements, obtained through imaging techniques like Particle Image Velocimetry (PIV) or catheterization. Simultaneous PV measurements are currently limited to invasive micro-catheter systems adapted from *in vivo* mouse experiments (Li et al., 2018; MacQueen et al., 2018; Kupfer et al., 2020; Mohammadi et al., 2022). Using an *in vivo* PV catheter raises concern about measurement accuracy in the absence of blood due to conductivity differences with cell culture medium and with tissue properties differing from the native myocardium (Chang et al., 2022). Moreover, PV catheters are often incompatible with electrical pacing, which is typically applied to standardize experimental conditions. Expanding non-invasive, multiplexed functional assessment capabilities therefore represents a key area for future development.

The highest performing model currently reports an EF of about 6%, well below physiological levels (50%–70%) (Choi et al., 2023). Nevertheless, reaching physiological levels is not essential in this application as long as functional parameters change as intended in a specific context-of-use (disease phenotype, response to drugs and treatment).

Ultimately, no existing platform excels equally in all dimensions. Finding the optimal balance between process efficiency and physiological complexity will be essential for the next-generation of cardiac chamber models. Modularity in design offers a promising strategy - allowing developers to tailor specific features to the intended setting. By aligning this trade-off in future models, the field is advancing toward systems that are not only suited for integration into academic and commercial drug testing and disease modeling pipelines but also positioned to make a tangible impact on patient care.
